# Use of comprehensive geriatric assessment among oncologists in Uruguay: perceptions, practices and barriers

**DOI:** 10.3332/ecancer.2026.2105

**Published:** 2026-03-31

**Authors:** Noelia Silveyraa, Clara Tambascob, Dahiana Amarilloc, Natalia Camejod, Gabriel Krygiere

**Affiliations:** Clinical Oncology Service, Hospital de Clínicas, Faculty of Medicine, University of the Republic, Montevideo, Uruguay; a https://orcid.org/0000-0002-0021-5760; b https://orcid.org/0000-0002-9771-7539; c https://orcid.org/0000-0002-8615-8639; d https://orcid.org/0000-0002-8684-0291; e https://orcid.org/0000-0002-0518-1854

**Keywords:** geriatric assessment, medical oncology, aged, neoplasm, health knowledge, attitudes, practice, surveys and questionnaires

## Abstract

**Introduction:**

Population ageing has increased the proportion of older adults with cancer, a group characterised by high comorbidity burden and marked clinical heterogeneity. Comprehensive Geriatric Assessment (CGA) is recommended by international guidelines to identify vulnerabilities and guide safer, more personalised treatment decisions. However, its implementation remains limited, and there is scarce information about its use in Uruguay.

**Objective:**

To describe perceptions, practices and barriers regarding the use of CGA among oncologists and trainees in Uruguay.

**Materials and Methods:**

We conducted an observational, cross-sectional study using an anonymous electronic questionnaire. Medical oncologists, radiation oncologists and physicians in training (postgraduate oncology training) involved in the clinical care of patients with cancer were included. A descriptive analysis was performed, and chi-square tests were used to compare frequencies between groups (*p* < 0.05).

**Results:**

A total of 60 physicians responded, representing approximately 60% of the national oncology community. Although 55% reported being familiar with the American Society of Clinical Oncology guideline, only 31.7% reported using formal CGA tools, and just 11.7% applied CGA routinely. Informal evaluation based on clinical judgement predominated. Functional status was the most frequently assessed domain (79%), while physical performance was the least used (>70% never or almost never). Participants familiar with the guideline assessed life expectancy and cognition more frequently, with statistically significant differences (p = 0.048). The most commonly reported barriers were lack of resources for referral, limited physical space and insufficient training. Among those unfamiliar with the guideline, perceived limited evidence and uncertainty about which tool to use were predominant barriers.

**Conclusions:**

CGA use in Uruguay is low, revealing a substantial gap between knowledge and implementation. The identified barriers highlight the need to strengthen training, improve available resources and promote institutional strategies to support CGA integration, with the aim of improving care for older adults with cancer.

## Introduction

Population ageing is a global phenomenon: it is estimated that the proportion of older people will increase from the current 8% to more than 16% in 2050 [1]. Uruguay is experiencing a marked process of population ageing: according to the results of the 2023 Census, 18.8% of the population are aged 65 or over. Life expectancy at birth for the period 2022–2023 is between 78.3 and 78.8 years [2]. This demographic change is associated with an increase in non-communicable diseases as the leading cause of morbidity and mortality, including c r and oncological pathologies [3, 4]. The National Cancer Registry shows that the highest incidence rates are concentrated in people over 80 years of age, both in men and women [5].

Older adults with cancer often have comorbidities and conditions that impact the tolerance and efficacy of cancer-specific treatments: 80% have at least one comorbidity, 27% are obese and 38% have some degree of disability [6]. Despite this, they continue to be underrepresented in clinical trials, which limits the evidence available to guide therapeutic decisions and often forces the extrapolation of data from younger, healthier populations [7].

In view of this situation, various organisations – including the American Society of Clinical Oncology (ASCO) and the FDA – have recommended reviewing trial inclusion criteria, improving the characterisation of older adults and promoting the systematic implementation of Comprehensive Geriatric Assessment (CGA) in oncology [8]. In 2018, ASCO published its first specific guidelines for this age group, with the aim of identifying vulnerabilities not detected in routine clinical practice and optimising therapeutic decision-making [9]. Despite the available evidence and international recommendations, the implementation of CGA in older adults with cancer remains low. The main barriers identified include lack of time in clinical practice, shortage of trained personnel and limited resources, which hinder the systematic identification of vulnerabilities and, consequently, personalised therapeutic decision-making [10].

In Uruguay, research conducted at the Hospital de Clínicas showed that the incorporation of the G8 test and GIV changed the initial indication for chemotherapy in 38% of patients ≥70 years of age, confirming its clinical value [11]. Despite the available evidence, the actual use of the IGA by oncologists in our setting is unknown. Therefore, the objective of this study is to evaluate the perspective of oncologists and physicians in training regarding the use of CGA, describe their patterns of use and identify barriers to its implementation.

### Study design

An observational, analytical, cross-sectional study was conducted, targeting professionals working in oncology care with older adult patients. The study was conducted between November 2021 and February 2022.

## Materials and methods

### Data collection

The information was obtained through an anonymous electronic questionnaire administered via the *SurveyMonkey* platform. The instrument consisted of three sections:

1. Informed consent.
2. Epidemiological survey (Appendix 1).
3. Questionnaire on the use of VGI in daily clinical practice (Appendix 2).

The invitation to participate was sent by email through the Uruguayan Society of Medical and Paediatric Oncology (SOMPU) and through instant messaging groups (*WhatsApp*) in the oncology community.

The questionnaire used was adapted from the instrument developed by Dale *et al* [10, 12] which assesses patterns of practice related to the formal and informal use of IGV in patients over 65 years of age.

- Formal GIV: use of validated tools to assess specific domains (functionality, cognition, nutrition and so on).
- Informal GIV: non-standardised clinical judgements based on observation or interaction with the patient.

The questionnaire included:

- Characteristics of respondents (professional title, place of work, care setting, clinical areas of greatest dedication and years of experience).
- Knowledge of the ASCO geriatric oncology guidelines (yes/no).
- Differences in the assessment and treatment of older versus younger patients.
- Frequency of formal VGI use according to a five-category Likert scale (always, most of the time, sometimes, rarely, never).
- Perceived barriers to implementing VGI in daily practice.

### Study population

The target population included medical oncologists, radiotherapists and doctors in training (postgraduate oncology) from both specialties who work with cancer patients in Uruguay. In the Uruguayan healthcare context, systemic cancer treatment is the exclusive responsibility of medical oncology, while radiation oncology does not administer this type of treatment. Only participants who gave their informed consent and were in contact with cancer patients were included. The questionnaire was distributed among SOMPU members and disseminated through informal networks (WhatsApp). No incentives were offered for participation. The estimated response time in the pilot test was 10 minutes.

### Statistical analysis

The statistical analysis consisted of a summary description of the responses obtained in the survey. To assess the frequency of VGI use, the five categories of the Likert scale were grouped into three levels: high frequency (‘always’ and ‘most of the time’), intermediate frequency (‘sometimes’) and low frequency (‘rarely’ and ‘never’). The differences between the different groups of respondents were analysed using the chi-square test (*χ*²), both for the grouped variables on the Likert scale and for the dichotomous variables (yes/no). Comparisons with a *p*-value < 0.05 were considered statistically significant.

### Ethical aspects

The study was conducted in accordance with national and international ethical standards applicable to research involving human subjects, including Executive Decree No. 379/008, Law 18.331 on Personal Data Protection, MERCOSUR regulations for clinical studies and the principles of the 2010 version of the Declaration of Helsinki. The questionnaire used was completely anonymous, and no data that could identify the participants was collected. The project was also reviewed and approved by the Research Ethics Committee of the Hospital de Clínicas, ensuring compliance with the relevant ethical standards.

## Results

### Population characteristics

Of the 60 physicians who responded to the survey, 63% work as specialists in clinical oncology, while 4% work in radiation oncology. Thirty-three percent are physicians in training (postgraduate studies in oncology) in both specialties. 63.3% provide daily care in public or private institutions, and 86.7% practise in Montevideo. Fifty-five percent were aware of the ASCO guidelines (Table 1).

### Geriatric assessment in daily clinical practice

Forty-five percent of participants reported that they make decisions differently for older adults than for younger patients, while 13% indicated that they never or almost never do so. This difference varies according to the workplace: most professionals in the interior reported that they ‘almost never’ modify their approach, unlike those working in Montevideo, who do so more frequently (Table 2).

In the academic setting, 70% said they evaluate older adults differently (‘almost always’ or ‘sometimes’), a proportion similar to that observed in the private or community setting (78%) (Table 2).

Only 31.7% use a formal assessment to evaluate older adults with cancer; 68% rely on an informal assessment or their clinical judgement. Regarding the frequency of use of formal GIV, 41.7% of respondents reported using it sometimes, 11.7% most of the time, while 28.3% indicated that they almost never use it and 18.3% never use it.

#### Clinical practice patterns based on knowledge of the ASCO guidelines

Fifty-five percent of respondents reported being familiar with the ASCO guidelines, with significant differences according to professional title (68.4% of specialists versus 30% of doctors in training (postgraduate oncology); *p*=0.02). No statistically significant differences were found according to workplace. In the academic setting, 55% were not familiar with the guidelines, while in the private or community setting, 61% were familiar with them (Table 2).

Those who were familiar with the guidelines reported more frequently evaluating older adults differently than younger patients (60% versus 40%) and using formal decision-making tools (63.2% versus 36.8%) (Table 3).

With regard to the domains of the VGI, life expectancy was the most frequently assessed among those familiar with the guidelines, with a statistically significant difference compared to those unfamiliar with them (100% versus 0%; *p* = 0.048). Significant differences were also observed in the assessment of cognition (81.8% versus 18.2%; *p* = 0.048). Although not statistically significant, in the other domains, the population familiar with the guidelines was found to assess the potential toxicity of chemotherapy, mood and falls more frequently, while weight loss was the domain least assessed by this group.

#### Use of validated QOL tools

The use of validated QOL tools was assessed in the domains of functional status, nutrition, social support, physical performance and cognition. The most widely used domain was functional status (79%), while the least evaluated was physical performance, with more than 70% stating that they never or almost never used it (Table 4).

Those familiar with the ASCO guidelines reported greater use of validated tools both before and after starting systemic treatment. Before treatment, the domain most valued by this group was cognition (100%), while those unfamiliar with the guidelines most frequently assessed social support (Table 4).

After starting treatment, professionals familiar with the guidelines continued to evaluate cognition and nutritional status more frequently, in contrast to those unfamiliar with the guidelines, for whom social support was the most frequently used domain (Table 4).

It should be noted that the percentages were calculated within each subgroup according to knowledge of the ASCO guidelines, and that the denominators may vary between domains due to incomplete responses to some questions.

### Barriers to performing VGI in clinical oncology practice

Regarding the barriers to performing CGA, the most commonly cited were the lack of resources available to refer these patients, lack of time and lack of training and/or education in CGA. Twenty-one percent of respondents considered that the current evidence for the use of CGA in routine oncology practice is limited, while 28% had no opinion on the matter. As for the other barriers considered (poor training, limited space for assessment and uncertainty about which GI assessment tool to use), nearly 40% of respondents reported experiencing them. In contrast, lack of financial reimbursement and lack of support staff were among the least frequently mentioned barriers (Table 5).

When these variables are considered according to knowledge of the ASCO guidelines, it can be seen that those who are familiar with the guidelines report that the existing barriers to performing VGI are the absence of support equipment, limited space and the lack of available resources to refer patients. However, the least frequently identified barriers were limited evidence for VGI and the lack of training and/or education. Among those who reported not knowing the guidelines, the most frequently cited barriers were limited evidence and uncertainty about which tool to use.

## Discussion

GIV is a central recommendation in oncogeriatrics since the 2018 ASCO guidelines, reinforced by updates that highlight its usefulness in detecting vulnerabilities not identified in routine oncological assessment and guiding safer and more personalised therapeutic decisions [12, 13]. Despite this support, its implementation remains limited globally due to barriers such as lack of time, shortage of trained personnel, absence of interdisciplinary teams and uncertainty about which tools to use, especially in resource-constrained settings [12–15].

This study, which included 60 physicians – approximately 60% of the national oncology community – offers a representative view of current practices in Uruguay. Although more than half of the participants report being familiar with the ASCO guidelines and 45% say they modify their approach in older adults, the systematic use of VGI is very low: only 11% use it routinely. Instead, informal assessment based on clinical judgement predominates, a gap between knowledge and implementation that has been widely documented in other countries and is often related to limitations in training and insufficient perception of usefulness [10, 12, 16].

Given that data collection took place between 2021 and 2022, our findings should be interpreted in the context of the guidelines in force at that time. Subsequently, the update of the ASCO 2023 guideline [12] and the recent ASCO 2025 global guideline [17] have reinforced the need not only to perform NGS, but also to ensure the implementation of guided interventions, even in resource- n environments. In this regard, the low adoption observed in our survey and the barriers identified anticipate the challenges that these most recent recommendations seek to address, particularly in non-academic settings or outside large urban centres.

The low level of knowledge of the ASCO geriatric oncology guidelines among trainee doctors, compared to oncology specialists, is striking. This finding could be explained, at least in part, by the absence of specific content on geriatric oncology and CGA in the national postgraduate programmes in force at the time the survey was conducted. In fact, the medical oncology training programme in force during the study period (2012) did not explicitly include this content. It should be noted that, following the study, the postgraduate programme was updated (2023), incorporating a greater emphasis on a comprehensive approach to the patient and aspects related to ageing, which could contribute to improving training in geriatric oncology in future cohorts.

A particularly relevant aspect of our results is the variation in the approach to older adults depending on the workplace. Doctors practising in Montevideo reported more frequently modifying their management (11% *always* and 38.5% *most of the time*), while in the interior, the predominant responses were ‘sometimes’ (37.5%) and ‘almost never’ (37.5%). These differences could be explained by structural and organisational factors already described in the literature, where it is observed that urban environments tend to have greater availability of multidisciplinary teams, access to geriatric resources, more robust diagnostic infrastructure and greater exposure to continuing education and updated guidelines, which favours the individualisation of treatment in older adults [18]. In contrast, rural settings tend to face limitations in human and material resources, lower availability of specialists, and logistical barriers that may restrict the possibility of adapting treatments according to frailty, comorbidities or overall function [19]. This regional variability in clinical practice reflects differences in healthcare culture, professional networks and operational capacity and highlights the need for specific strategies to improve the implementation of VGI outside urban centres.

Differences were also observed depending on the work environment. Professionals in academic centres reported adapting their management in older adults more frequently: 40.9% do so ‘most of the time’ and 36.4% ‘sometimes’, while only 13.6% indicated ‘almost never’. In contrast, in community or private practice, adaptation was also frequent, with comparable values (34.2% and 44.7%, respectively), and a low percentage reporting ‘almost never’ (7.9%).

These differences can be explained by factors previously described in the literature. In the academic setting, there is greater exposure to evidence-based medicine, access to multidisciplinary teams and participation in clinical research, which favours the integration of international recommendations and the use of VGI to personalise treatment for older adults [20]. In the private sector, care practices equally oriented towards treatment adaptation have been described, although in some contexts with less availability of interdisciplinary resources or structural support to facilitate more complex assessments such as VGI [21].

Taken together, these findings suggest that the academic environment can act as a facilitator for the adoption of geriatric oncology practices, while the private sector could benefit from strategies aimed at strengthening available structural support and facilitating the implementation of GIV when necessary.

Regarding the use of formal tools, our results show that those who are familiar with the ASCO guidelines not only report evaluating older adults more frequently in a differentiated manner, but also use validated instruments more frequently before and after initiating systemic treatment.

These findings are consistent with those reported in international studies, which show an association between knowledge of the ASCO guidelines and greater use of GI assessment tools; however, this greater adherence does not necessarily translate into full and systematic implementation.

The main barriers identified to achieving comprehensive implementation – lack of time, staff shortages and organisational limitations – persist regardless of the level of knowledge, as also noted in the ASCO 2023 guideline update [12, 13]. This reinforces that knowledge of the guideline alone does not guarantee its systematic implementation.

The uneven use of specific domains is also a point of interest. Functional status was the most frequently assessed domain, which is consistent with its integration into routine oncology practice. However, physical performance was the least used, despite its prognostic relevance. Similarly, cognition was widely assessed by those who were familiar with the guidelines, but very little by those who were not, replicating international patterns where this domain remains underestimated despite its strong impact on toxicity, adherence and survival [6, 14]. Social support, on the other hand, was assessed more frequently by the group unfamiliar with the guidelines, which could reflect an intuitive rather than a methodological approach.

The clinical relevance of these results is evident: evidence shows that the prognosis of older adults with cancer is mainly determined by comorbidities and physical, biological and cognitive limitations – factors that are not adequately captured by conventional tools such as ECOG – and that a structured VGI allows for accurate identification [6, 9, 12, 14]. These vulnerabilities are associated with an increased risk of toxicity, unplanned hospitalisations, poorer treatment tolerance and lower survival, while functional and nutritional status are independent prognostic factors and comorbidities predict non-cancer-related mortality [6, 14, 18]. Furthermore, early detection of functional or cognitive impairments has been linked to lower rates of prolonged hospitalisation, especially in haematological tumours [18].

Finally, the analysis of barriers provides key information for understanding the low adoption of GI in our practice. These findings are consistent with those reported in other countries in the region. For example, in a study conducted in Mexico [23], the systematic implementation of geriatric assessment was low and the main barriers identified included a lack of trained personnel, limited specific knowledge and time constraints, reinforcing the regional nature of these difficulties. In the general population surveyed, the most frequent barriers were the lack of resources to refer patients (51.4% agreed), limited physical space (66.7% ‘strongly agreed’ among those familiar with the guideline), and the absence of specific training or education (50% ‘strongly agreed’). However, when stratified by knowledge of the ASCO guidelines, differential patterns emerge that are particularly relevant.

Among those familiar with the guidelines, the main barriers reported were the lack of support equipment (66.7%) and limited physical space (66.7%). This suggests that, in this group, the main obstacle is not conceptual but structural: even though the clinical value of VGI is recognised, the lack of human resources and infrastructure prevents its systematic application. This situation coincides with what has been described in the literature, where the implementation of VGI is limited by the lack of trained personnel, interdisciplinary teams and organisational systems that allow for more extensive assessments [12, 22, 24].

In contrast, those unfamiliar with the guideline reported insufficient evidence (66.7% ‘strongly agree’) and uncertainty about which tool to use (58.8%) as the predominant barriers, reflecting a low familiarity with the domains and methods of VGI. This pattern mirrors that observed in non-academic settings or those with less access to continuing education, where the perception of complexity, lack of clarity regarding the available instruments and absence of systematic training generate resistance and hinder the integration of VGI into the usual clinical workflow [24].

The literature also points out that, in these contexts, the lack of institutional leadership or leading professionals to promote the use of GI, combined with organisational priorities focused on healthcare efficiency, contributes to relegating GI to a secondary role [23–25]. Overall, our findings show that the gap in the implementation of VGI is not limited to a lack of knowledge, but includes structural, organisational and training barriers that require specific interventions to be overcome.

On the other hand, evidence suggests that the clinical impact of GIV does not depend solely on its implementation, but also on who implements the interventions derived from the assessment. The most effective models are those based on multidisciplinary teams, with the participation of geriatricians and other health professionals, who facilitate the translation of GIE findings into concrete and sustained management plans [15, 26]. In the absence of these teams, the implementation of recommendations is often incomplete, even when the geriatric assessment has been performed, limiting its clinical benefit [1–3]. This reality is particularly relevant in resource-constrained settings, where the availability of specialists and formal referral pathways remains a challenge [14, 27].

Taken together, these data point to a concrete opportunity for improvement in national oncology practice. The systematic implementation of GI assessment would allow for safer and more personalised therapeutic decisions, reduce toxicities and promote better clinical outcomes, in line with current oncogeriatric recommendations [6, 9, 12, 14].

Among the strengths of this study is the inclusion of a large proportion of the national oncology community, which offers a representative view of practices and perceptions of VGI in Uruguay. In addition, the use of a questionnaire based on a previously validated instrument facilitates comparison with international studies and provides new and relevant information to guide improvement strategies in oncogeriatrics.

However, the study has some limitations. First, the self-reported nature of the survey may introduce memory or social desirability biases and voluntary sampling may favour the participation of professionals who are more interested in the subject. Furthermore, the cross-sectional design does not allow for an evaluation of the quality of GI use or its clinical impact, limiting itself to describing perceptions and reported frequency.

## Conclusion

This study provides a first national approximation of the use of CGA among oncologists in Uruguay and reveals a significant gap between recognition of its usefulness and its actual application in clinical practice. Although more than half of the professionals claim to be familiar with the ASCO guidelines, the systematic use of formal tools is low and informal assessment predominates, with marked differences depending on the location and work environment. Likewise, the barriers identified – both structural and educational – highlight the need to strengthen resources, training and institutional support to facilitate the implementation of CGA.

The findings of this study underscore the opportunity and urgency of more robustly integrating VGI into cancer care for older adults in Uruguay. Moving in this direction would improve the identification of vulnerabilities, optimise therapeutic decision-making and contribute to safer and more personalised clinical outcomes for a growing and particularly vulnerable population.

## Conflicts of interest

The authors declare no conflicts of interest.

## Funding

The study received no funding.

## Figures and Tables

**Table 1.** Demographic characteristics.

Epidemiological variables	*N* (%)
Primary professional qualification	
Doctor of Medicine, postgraduate degree in clinical oncology/radiotherapy	20 (33%)
Specialist in clinical oncology	38 (63%)
Specialist in radiation oncology	2 (4%)
Work environment	
Academic	22 (36.7%)
Community or private practice	38 (63.3%)
Place of work	
Inland	8 (13.3%)
Montevideo	52 (86.7%)
Knowledge of ASCO guidelines	
Yes	33 (55%)
No	27 (45%)

**Table 2.** When making management decisions for your patients aged 65 and over, how often do you assess them differently compared to your younger patients?

Variable	Category	Always (%)	Most of the time (%)	Sometimes (%)	Almost never (%)	Never (%)
Workplace	Inland	0	25	37.5	37.5	0
	Montevideo	11	38.5	5.8	42.3	3.8
Work environment	Academic centre	0	40.9	36.4	13.6	0
	Community or private practice	0	34.2	44.7	7.9	0

**Table 3.** Are you familiar with the ASCO geriatric oncology guideline: Practical assessment and management of vulnerabilities in older patients receiving chemotherapy [9]?

Variable	Category	Total n (%)	Not familiar with the guidelines n (%)	Familiar with guidelines n (%)
A. Primary qualification				
	Doctors in training (postgraduate degree in clinical oncology/radiotherapy)	20 (33.3)	14 (70.0)	6 (30.0)
	Specialist in clinical oncology	38 (63.3)	12 (31.6)	26 (68.4)
	Radiation oncology specialist	2 (3.3)	1 (50.0)	1 (50.0)
B. Place of work				
	Inland	8 (13.3)	2 (25.0)	6 (75.0)
	Montevideo	52 (86.7)	25 (48.1)	27 (51.9)
C. Work environment				
	Academic centre	22 (36.7)	12 (54.5)	10 (45.5)
	Community/private practice	38 (63.3)	15 (39.5)	23 (60.5)
D. Different assessment frequency in ≥65 years				
	Always	5 (8.3)	2 (40.0)	3 (60.0)
	Most of the time	11 (18.3)	4 (36.4)	7 (63.6)
	Sometimes	25 (41.7)	12 (48.0)	13 (52.0)
	Almost never	18 (30.0)	6 (33.3)	12 (66.7)
	Never	1 (1.7)	1 (100.0)	0 (0.0)
E. Type of assessment used				
	Informal, based on clinical judgement	41 (68.3)	21 (51.2)	20 (48.8)
	Formal, with validated tools	19 (31.7)	7 (36.8)	12 (63.2)

**Table 4.** Frequency of use of specific tools to assess the different domains of QOL before and after starting systemic treatment, according to knowledge or lack thereof of the ASCO guidelines.

VGI domains		Use of VGI domains BEFORE starting systemic treatment		Use of VGI domains AFTER starting systemic treatment	
		**Are you familiar with the ASCO guidelines?**		**Are you familiar with the ASCO guidelines?**	
		**No**	**Yes (%)**	**No (%)**	**Yes (%)**
Functional status	Always	38.9	61.1	50	50
	Most of the time	37.5	62.5	37.5	62.5
	Sometimes	53.8	46.2	50	50
	Almost never	33.3	66.7	14.3	85.7
	Never	58.3	41.7	53.3	46.7
Physical condition	Always	33.3	66.7	50	50
	Most of the time	33.3	66.7	33.3	66.7
	Sometimes	33.3	66.7	33.3	66.7
	Almost never	21.4	78.6	21.4	78.6
	Never	60	40	60	40
Social support	Always	40	60	40	60
	Most of the time	50	50	50	50
	Sometimes	22.2	77.8	22.2	77.8
	Almost never	44.4	55.6	44.4	55.6
	Never	51.5	48.5	51.5	48.5

Note: Percentages were calculated within each subgroup according to knowledge of the ASCO guidelines; denominators may vary between domains due to incomplete responses to some questions.

**Table 5.** Perceived barriers to implementing VGI in clinical practice (*n* = 60).

Barrier	Strongly agree *n* (%)	Agree *n* (%)	Disagree *n* (%)	Strongly disagree *n* (%)	No opinion *n* (%)
Insufficient time	31 (51.7)	23 (38.3)	4 (6.7)	1 (1.7)	1 (1.7)
Lack of resources to refer patients	8 (13.3)	35 (58.3)	12 (20.0)	1 (1.7)	4 (6.7)
Lack of training/education in VGI	28 (46.7)	25 (41.7)	1 (1.7)	1 (1.7)	5 (8.3)
Limited evidence for the use of VGI	13 (21.7)	19 (31.7)	11 (18.3)	0	17 (28.3)
Uncertainty about which tool to use	24 (40.0)	19 (31.7)	9 (15.0)	0	8 (13.3)
Limited space for assessment	20 (33.3)	22 (36.7)	10 (16.7)	1 (1.7)	7 (11.7)
Lack of financial reimbursement	4 (6.7)	9 (15.0)	27 (45.0)	8 (13.3)	12 (20.0)
Lack of support equipment	7 (11.7)	7 (11.7)	35 (58.3)	10 (16.7)	1 (1.7)

## Appendix 1. Sociodemographic survey.

1. Do you treat older adults with cancer in your regular clinical practice?
  Yes
  No (The answer displays a disqualified message)
2. What is your primary professional qualification?
  Doctor of Medicine, doctors in training (postgraduate in oncology) in oncology (clinical/radiotherapy)
  Clinical Oncology Specialist
  Specialist in Radiation Oncology.
3. What are the most common types of oncological pathologies you see in your daily practice? (Do not select more than three answers)
  CNS cancer
  Head and neck cancer
  Lung cancer
  Mesothelioma
  Oesophageal cancer
  Stomach cancer
  Gastrointestinal cancer
  Colorectal cancer
  Bile duct/gallbladder cancer
  Pancreatic cancer
  Hepatocellular carcinoma
  Breast cancer
  Cervical cancer
  Bladder cancer
  Kidney cancer
  Prostate cancer
  Testicular cancer
  Melanoma
  Sarcoma
  Other, please specify
4. How would you describe your primary work environment?
  Academic Medical Centre/University
  Community or private practice (in office/hospital)
5. Where do you perform most of your work?
  Montevideo
  Inland

## Appendix 2. Questionnaire on CGA in daily practice.

Questions 1–8 ask whether you assess and manage patients over 65 differently from younger patients, and if so, how. The questions specifically ask how often, when and how you assess older patients, whether you use standardised processes/tools or less formal approaches and the possible barriers to such assessments.

1. Are you familiar with the ASCO Geriatric Oncology Guideline: Practical Assessment and Management of Vulnerabilities in OlderPatients Receiving Chemotherapy [9]?
  Yes
  No
2. When making management decisions for your patients aged 65 years or older, how often do you assess them differently comparedto your younger patients?
  Always
  Most of the time
  Sometimes
  Almost never
  Never
3. When making management decisions for your patients over the age of 65, how do you assess these older patients differently fromyour younger patients?
  Formally, with specific validated tools
  Informally, based on my own judgement
  Not at all
4. For your patients aged 65 and older, how often do you perform a multidimensional geriatric assessment using validated tools?
  Always
  Most of the time
  Sometimes
  Almost never
  Never
5. In the last 12 months, have you used validated tools to assess the following domains in patients aged 65 years or older? Check all that apply and list any additional tools you use under ‘other’.
  Functional status: (e.g., instrumental activities of daily living (IADL), Barthel Index)
  Comorbidity: (e.g., Charlson Comorbidity Index, CIRS-G or HCT-Cl)
  Mood: (e.g., Yessavage Geriatric Depression Scale)
  Cognition: (e.g., Mini Mental State Examination (MMSE), Pfeffer scale)
  Risk of chemotherapy toxicity: (e.g., Cancer and Aging Research Group (CARG) or Chemotherapy Risk Assessment Scale for High-Age Patients (CRASH))
  Detection of non-cancer-related mortality risk: such as Geriatric-8 (G8) or Vulnerable Elders Survey-13 (VES-13)
  Falls: (e.g., ask about the number of falls in the last 6 months or since the last visit)
  Calculated unintentional weight loss: (e.g., check weight and document unintentional weight loss of 5% of body weight, mini nutritional assessment (MNA))
  Life expectancy: (e.g., ePrognosis.com)
  Other, please list:
  Instructions: Questions 6 and 7 are about when you perform these assessments. They may appear identical to the previous ones, but they are different. The specific tools listed are examples.
  Before initiating systemic therapy in your patients aged 65 years or older, how often do you use specific tools to:

**Table:**

	Always	Most of the time	Sometimes	Almost never	Never
Assess a patient's functional status beyond KPS and ECOG? (e.g., instrumental activities of daily living (IADL), Barthel Index)					
Assess a patient's physical status? (e.g., Short Physical Performance Battery (SPPB), walking speed)					
Assess a patient's social activity/support? (e.g., Gijón scale, Medical Outcomes Study Social Support Survey (MOS-SS))					
Assessing the nutritional status of a patient? (e.g., mini nutritional assessment (MNA))					
Assessing a patient's cognitive level? (e.g., MMSE)					
Other, please specify					

0. After an initial assessment of your patients aged 65 and over, when there is a specific change in a patient’s disease status (i.e., disease progression, significant treatment toxicity), how often do you use these tools to inform management decisions?

**Table:**

	Always	Most of the time time	Sometimes	Almost never	Never
Assess a patient's functional status beyond KPS and ECOG? (e.g., instrumental activities of daily living (IADL), Barthel Index)					
Assess a patient's physical status? (e.g., Short Physical Performance Battery (SPPB), walking speed)					
Assess a patient's social activity/support? (e.g., Gijón scale, Medical Outcomes Study Social Support Survey (MOS-SS))					
Assessing a patient's nutritional status? (e.g., Mini Nutritional Assessment (MNA))					
Assess a patient's cognitive level? (e.g., MMSE)					
Other, please specify					

0. Indicate your level of agreement regarding BARRIERS to performing a geriatric assessment for your patients aged 65 and over:

**Table:**

	Strongly agree	Somewhat agree	Somewhat agree	Disagree	Strongly disagree
Insufficient time					
Limited or no financial reimbursement none to the supplier					
Lack of support staff					
Lack of training, skills, knowledge or experience in EGI					
Uncertainty about which assessment tools to use					
Limited or unavailable space to conduct the assessment (i.e., consulting room and so on)					
Lack of available resources to refer such patients					
Limited evidence to support their use in practice					
Other, please specify					
